# Inconsistent condom use among men who have sex with men in Brazil: the role of trusting the sexual partner

**DOI:** 10.1590/1980-549720260052

**Published:** 2026-10-05

**Authors:** Jonatan da Rosa Pereira da Silva, Rafael Steffens Martins, Afonso Valau de Lima, Andrea Fachel Leal, Laio Magno, Ines Dourado, Maria Amélia de Sousa Mascena Veras, Carl Kendall, Ligia Regina Sansigolo Kerr, Daniela Riva Knauth

**Affiliations:** I Universidade Federal do Rio Grande do Sul, Department of Social Medicine - Porto Alegre (RS), Brazil.; II Universidade do Extremo Sul Catarinense, Socioeconomic Development and Innovation Observatory - Criciúma (SC), Brazil.; III Universidade Federal do Rio Grande do Sul, Department Sociology - Porto Alegre (RS), Brazil.; IV Universidade do Estado da Bahia, Department of Public Health - Salvador (BA), Brazil.; V Universidade Federal da Bahia, Department of Public Health - Salvador (BA), Brazil.; VI Faculty of Medical Sciences of Santa Casa de São Paulo - São Paulo (SP), Brazil.; VII Tulane University, Celia Scott Weatherhead School of Public Health and Tropical Medicine - Louisiana, United States.; VIII Universidade Federal do Ceará, Department of Public Health - Fortaleza (CE), Brazil.

**Keywords:** Sexual and gender minorities, Sexual behavior, Condoms, Trust, HIV, Minorias Sexuais e de Gênero, Comportamento Sexual, Preservativos, Confiança, HIV

## Abstract

**Objective:**

To analyze the statistical association of trust in a sexual partner and inconsistent condom use among men who have sex with men in Brazil.

**Methods:**

Data came from a nationwide biological and behavioral surveillance cross-sectional study conducted in 2016 across 12 Brazilian cities with 4,176 men who have sex with men, utilizing respondent-driven sampling. This analysis focused on 2,010 men who have sex with men. We analyzed the data using Poisson Regression with robust standard errors. Weights and design were incorporated.

**Results:**

Almost half of the men who have sex with men had inconsistent condom use during their last anal intercourse (48.9%, n=880). Inconsistent condom use was more prevalent among men who have sex with men who said they accept having sex without using a condom when trusting their sexual partner (84.2%, p<0.0001). Trust in a sexual partner had a strong association with inconsistent condom use during last anal intercourse among all men who have sex with men (adjusted prevalence ratio (PRa) of 1.73, 95%CI 1.25-2.40, p<0.0001). This association remains statistically significant in our stratified analysis among single/separated/widowed men who have sex with men (PRa=1.93, 95%CI 1.44-2.60, p<0.0001) and among men who have sex with men who are married/live together (PRa=1.31, 95%CI 1.11-1.77, p=0.045), indicating that trust can be experienced in different relationship contexts, not only with main or steady partners, such as a husband or boyfriend.

**Conclusion:**

Trust plays an important role in sexual behavior among men who have sex with men, and it is related to a higher prevalence of inconsistent condom use. Since the HIV epidemic among men who have sex with men in Brazil appears to be growing, future prevention strategies have to consider this and incorporate trust into counseling actions because trusting relationships also carry a risk of HIV infection.

## RESUMO

**Objetivo:**

Mensurar a associação estatística entre a confiança no parceiro sexual e o uso inconsistente de preservativo (UIP) de homens que fazem sexo com homens (HSH) no Brasil.

**Métodos:**

Os dados analisados são provenientes de um estudo transversal com 4.176 HSH, conduzido em 2016, em 12 capitais brasileiras, utilizando a técnica *respondent-driven sampling* para o recrutamento. Para a análise, foram utilizados dados de 2.010 HSH. Pesos e *design* foram incorporados em todas as análises.

**Resultados:**

Quase metade dos HSH fez UIP na última relação sexual (48,9%, n=880). O UIP foi mais prevalente entre HSH que afirmaram que aceitavam fazer sexo sem usar preservativos quando confiavam no parceiro sexual (84,2%, p<0.0001). A confiança no parceiro sexual apresentou forte associação com o UIP entre todos os HSH analisados (razão de prevalência [RPa]=1,73, IC95% 1,25-2,40, p<0,0001). Essa associação permaneceu significativa em nossa análise estratificada entre HSH solteiros/separados/divorciados (RPa=1,93, IC95% 1,44-2,60, p<0,0001) e HSH casados/com companheiro (RPa=1,31, IC95% 1,11-1,77, p<0,0001), indicando que a confiança pode ser experenciada em diferentes relações, não somente com parceiros fixos como namorados ou maridos.

**Conclusão:**

A confiança no parceiro é um importante preditor para o comportamento sexual dos HSH e está associada ao UIP. Por causa do crescimento da epidemia de HIV entre HSH no Brasil, futuras ações em saúde precisam incorporar a confiança no parceiro em ações de aconselhamento em saúde, uma vez que o UIP é um fator de risco para a infecção pelo vírus da imunodeficiência humana (HIV) mesmo em relações em que existe confiança entre os parceiros.

## INTRODUCTION

Globally, men who have sex with men (MSM) still face a disproportionate burden of HIV infection. According to data from the World Health Organization (WHO), MSM are 26 times more likely to become infected with HIV over their lifetime compared to the general population^1^. Recent evidence also indicated that in 2022 MSM were responsible for 20% of new HIV cases among adults aged 15-49^2^. In Brazil, two national behavioral studies indicated that HIV prevalence among MSM increased from 10.5% in 2009 to 18.4% in 2016^3^ ^,^ ^4^. Despite the lack of recent nationwide estimates of HIV prevalence and incidence among Brazilian MSM, this population remains disproportionately affected by the HIV epidemic. According to the Brazilian Ministry of Health, men accounted for 65.9% (450,922) of the 683,395 HIV cases reported in Brazil between 1991 and September 2025. Furthermore, sex between men remains the leading reported mode of HIV exposure among male cases, reinforcing the disproportionate burden of HIV among Brazilian MSM^5^.

Previous literature on sexual behavior facing the HIV epidemic among MSM highlighted the role of inconsistent condom use (ICU) and factors associated with this behavior on new infections. However, this research focused mainly on sociodemographic (age, race, schooling, and socio-economic status)^6^ ^,^ ^7^ ^,^ ^8^ ^,^ ^9^ and structural factors (sexual orientation discrimination and access to health services)^10^ ^,^ ^11^ ^,^ ^12^ associated with ICU. On the other hand, studies addressing the emotional dimensions of sexual behavior among MSM have received less attention and, when conducted, have primarily focused on relationships with regular partners, such as boyfriends or husbands^13^ ^,^ ^14^ ^,^ ^15^.

The scientific study of trust emerged in the 1950s and 1960s across multiple disciplines, including philosophy, sociology, behavioral economics, social psychology, political science, and neurobiology. These contributions established trust as a multidimensional construct involving both emotional and cognitive processes that shape interactions between individuals. Over time, the concept of trust has been incorporated into research on interpersonal relationships and sexual health, given that sexual interactions are inherently social and may be influenced by perceptions of trust between partners. Thus, understanding trust may provide important insights into how individuals perceive and manage sexual risks, including decisions regarding condom use^13^.

A longitudinal qualitative study in the United States highlighted the role of emotional context in sexual behavior. MSM who experienced a deep level of love, trust, and intimacy with their partners feel less need to use a condom since they believe that these relationships carry less sexual risk of HIV infection or sexually transmitted infections^15^. Another study in South Africa indicated that MSM who report a high frequency of past ICU were more likely to believe that it is not necessary to use condoms with a trusted or steady sexual partner regardless of their current partnership status^14^.

This evidence may argue that MSM did not make decisions about their sexual behavior in an emotional vacuum, and trust in a sexual partner may influence their perception of risk. However, new studies with different approaches are needed to evaluate the role of trust in ICU among MSM and expand the analysis with other sexual partners in addition to regular partners, especially in Brazil, where this hypothesis has not been tested quantitatively.

This study aims to analyze the association of trust in a sexual partner with ICU among MSM in Brazil.

## METHODS

### Study location, design, population, and sample

The study is based on data collected in a behavioral and biological surveillance study entitled “A Nationwide Study of the Behaviors, Attitudes, Practices, and Prevalence of HIV, Syphilis, and Hepatitis B and C among MSM in Brazil”, conducted from June to December 2016 in 12 state capitals: Manaus and Belém in the Northern region; Fortaleza, Recife, and Salvador, in the Northeast region; Brasília and Campo Grande in the Central-west; Belo Horizonte, Rio de Janeiro, and São Paulo in the Southeast; and Curitiba and Porto Alegre in the South.

The sample consisted of men defined as biologically male, 18 years of age or older, reporting oral sex or anal intercourse with another man in the previous 12 months; and living, studying, or working in one of the study cities.

A total of 4,176 MSM were enrolled in the nationwide study. Of these, 2,018 reported never engaging in condomless sex and were therefore not eligible for the subsequent questions on reasons for condomless sex. The remaining 2,158 MSM constituted the eligible population for the present analysis, which examined the association between trust in a sexual partner and ICU.

### Data collection

We used respondent-driven sampling (RDS) to recruit participants. Before the survey, we conducted formative qualitative research using individual interviews and focus group discussions to explore participation in the study, including willingness to recruit, HIV and syphilis testing, and study logistics, such as level and kind of incentive, siting, and working hours.

To start recruiting, we purposively selected six MSM in each city. These individuals, known as seeds, had large social networks of MSM and represented different sociodemographic characteristics. Next, each of these seeds invited three other MSM from their social networks using three invitation coupons. This procedure was repeated in sequence until the desired sample size was reached, estimated a priori as 350 participants per city. Participants received BRL 25.00 (USD$ 7.40) for their participation and for each recruited participant (BRL 25.00 for each one up to three new participants - BRL 100.00) to reimburse their expenses for transportation and food. Data were collected using computer-assisted personal interviews (CAPI) at fixed office sites, where educational materials, condoms, and lube were offered, and counseling, blood draws, and results were provided. A total of 4,176 MSM were recruited in the 12 cities, who signed separate consent forms for the interview and testing. The Research Ethics Committee of Universidade Federal do Ceará approved the research project. Further details can be found in other publications^2^ ^,^ ^3^ ^,^ ^16^.

### Outcome

The outcome was ICU during last anal intercourse among MSM, measured by the question “*Thinking about your last anal intercourse, regardless of partner type and role (receptive or insertive), did you use a condom? (yes/no)*”. MSM who answered “no” were considered inconsistent condom users.

### Main exposure variable

The main exposure variable of our study was trust in a sexual partner, measured by the question “*When trusting a sexual partner, do you accept having sex without using a condom? (yes/no)*”.

### Other covariates

Age (18-24 years old, ≥25 years old), self-declared race (white, non-white (black, mixed, brow, Indigenous/native, and Asian origin), schooling (primary and incomplete secondary education, complete secondary education, and incomplete or complete undergraduate education), marital status (single/separated/widowed, married/living together), knowledge about HIV/AIDS (low, middle, high) measured by Item Response Theory (IRT), as described by Guimarães et al.^16^, self-perception of HIV infection risk (low, middle, high) measured using the question “How do you assess your risk of becoming infected with HIV at the moment?”, and medical appointment in the previous 12 months (yes, no).

### Statistical analysis

Dependence between observations resulting from recruitment chains in RDS, i.e., the probability of unequal selection and the different sizes of each participant’s contact network, was considered. Gile’s estimator was used to weigh the proportion estimates with a 95% confidence interval using RDS Analyst. We aggregated all data from the 12 cities into a single dataset with each city serving as a stratum. Statistical analyses were performed using Stata 12.0 (Stata Corporation, College Station, TX), through the survey command, which considers the effects of complex sampling and sample weights required by the use of RDS recruitment.

A descriptive analysis based on inconsistent or consistent condom use was performed. In this analysis, data were presented using relative and absolute frequencies, and the differences were evaluated by the Rao-Scott chi-square test (Table 1).

**Table 1.** Sample characteristics by inconsistent and consistent condom use among men who have sex with men, Brazil, 2016.

	Total sample (n=2,010)	Condom use during last sexual intercourse		p-value
		Inconsistent (48.9%, n=880)	Consistent (51.1%, n=1,130)	
Age (years old)				
18-24	61.8% (1,281)	60.7% (558)	62.9% (723)	0.676
≥25	38.2% (729)	39.3% (322)	37.1% (407)	
Race				
White	36% (694)	34.6% (298)	37.3% (396)	0.571
Non-white	64% (1,316)	65.4% (582)	62.7% (734)	
Schooling				
Primary education/incomplete secondary education	31% (467)	36.5% (220)	25.8% (247)	0.052
Complete secondary education/incomplete or complete undergraduate education	69% (1,543)	63.5% (660)	74.2% (883)	
Marital status				
Single/separated/widowed	83.9% (1,747)	76.4% (722)	91.1% (1,025)	0.001
Married/living together	16.1% (263)	23.6% (158)	8.9% (105)	
Accept to have sex without using a condom when they trust their sexual partner				
No	26.3% (547)	15.8 (178)	36.4% (369)	<0.0001
Yes	73.7% (1,463)	84.2% (702)	63.6% (761)	
Self-perception of HIV infection risk				
None/Low	66.2% (1,338)	58.3% (557)	73.7% (781)	0.001
Middle/High	33.8% (672)	41.7% (323)	26.3% (349)	
HIV knowledge				
High	25.8% (439)	29.2% (208)	22.6% (231)	0.077
Middle	47.3% (1,051)	41.7% (465)	52.7% (586)	
Low	26.9% (520)	29.1% (207)	24.7% (313)	
Medical appointments in previous 12 months				
Yes	78.2% (1,588)	75% (681)	81.3% (907)	0.200
No	21.8% (422)	25% (199)	18.7% (223)	

*Data presented by relative and absolute frequencies; **p-value associated with the Rao-Scott adjusted χ^2^ test.

Poisson Regression models with robust standard errors were used to evaluate the association of the outcome, ICU, with the main exposure variable, trust in a sexual partner (Table 2). We estimated an unadjusted model for the entire sample to measure the association between the outcome and the main exposure variable, obtaining the unadjusted prevalence ratio (PRu) and respective 95% confidence interval (95%CI) (column 2). Sequential adjustments were made for age, self-declared race, schooling, marital status (model 2), knowledge about HIV/AIDS, self-perception of HIV infection risk (model 3), and medical appointment (model 4).

**Table 2.** Association between trust in a sexual partner and inconsistent condom use during last sexual intercourse among men who have sex with men in Brazil.

Trust the sexual partner	Inconsistent condom use during last anal intercourse					
	Total sample		Single/separated/widowed MSM*		MSM who are married/live together	
	PR (95%CI)	p-value	PR (95%CI)	p-value	PR (95%CI)	p-value
Model 1						
No	Reference	0.001	Reference	<0.0001	Reference	0.0339
Yes	1.90 (1.34-2.71)		2.18 (1.60-2.97)		1.24 (0.78-1.98)	
Model 2*						
No	Reference	<0.0001	Reference	<0.0001	Reference	0.243
Yes	1.85 (1.34-2.54)		2.13 (1.58-2.89)		1.24 (0.85-1.82)	
Model 3						
No	Reference	<0.0001	Reference	<0.0001	Reference	0.048
Yes	1.74 (1.26-2.41)		1.94 (1.45-2.61)		1.30 (1.13-1.79)	
Model 4						
No	Reference	<0.0001	Reference	<0.0001	Reference	0.045
Yes	1.73 (1.25-2.40)		1.93 (1.44-2.60)		1.31 (1.11-1.77)	

Note: Total sample n=2,010; single/separated/widowed MSM n=1,747; MSM who are married/live together n=263. Model 1 - unadjusted; Model 2 - Model 1 + age, race, schooling, and marital status; Model 3 - Model 2 + self-perception of HIV infection risk and HIV knowledge; Model 4 - Model 3 + medical appointments in the last year; *for single/separated/widowed MSM and MSM who are married/live together, the variable marital status was not included in Model 2. p-value associated with the adjusted Wald test.

Considering the trust dynamics in relationships with main partners (such as boyfriend or husband), we performed a stratified analysis according to marital status to evaluate the association between trust in a sexual partner and ICU in these two scenarios: (single/separated/widowed MSM (Table 2, column 4) and MSM who are married/live together (Table 2, column 6). The same sequential adjustments were made for this analysis, and PR and CI were estimated.

## Data availability statement:

The complete dataset supporting the findings of this study is available from the corresponding author upon reasonable request.

## RESULTS

Among the 2,158 MSM, missing data were found in sociodemographic variables, so this analysis focused on 2,010 MSM. The prevalence of ICU during last anal intercourse was 48.9% (95%CI 44.3-53.5%, n=880).

Table 1 presents a descriptive analysis of the sample. Most MSM were younger (18-24 years old, 61.8%, 1,281), self-declared non-white (64%, 1,316), single/separated/widowed (84.1%, 1,661), and had a higher level of schooling (complete secondary/ incomplete or complete undergraduate education). Middle and low levels of HIV/AIDS knowledge were presented by 47.3% (1,051) and 26.9% (520) of MSM, respectively. Also, in terms of health services, most MSM had a medical appointment in the previous 12 months (78.2%, 1,588). We do not identify statistical differences between these variables and trust in a sexual partner to have sex without using a condom.

On the other hand, MSM who are married/live together (23.6%, p=0.001), with moderate/high risk perception (41.7%, p=0.001) had a higher frequency of ICU. ICU was also higher among MSM who accept having sex without using a condom when they trust their sexual partners (84.2%, 702, p<0.0001).

Table 2 presents the association between trust in a sexual partner and ICU during last anal intercourse. In our unadjusted model, with the entire sample, MSM who accept having sex without using a condom when trusting their sexual partner had a higher prevalence ratio of ICU (PRu 1.90, 95%CI 1.34-2.71, p=0.001). After adjustments by age, race/skin color, schooling, marital status (model 2), HIV risk perception, level of HIV knowledge (model 3), and medical appointment in the previous 12 months (model 4), this association remains strong (adjusted prevalence ratio (PRa) of 1.73, 95%CI 1.25-2.40, p<0.0001) (Table 2, column 2).

In our stratified analysis, even among single/separated/widowed MSM (1,747), trust in the sexual partner was associated with ICU during last anal intercourse (PRu=2.18, 95%CI 1.60-2.97, p<0.0001). After all sequential adjustments for sociodemographic variables (age, race/skin color, schooling), HIV risk perception, level of HIV knowledge, and health services variables (medical appointment), this association remains strongly significant. The PRa of ICU during last anal intercourse among single/separated/widowed MSM who stated they accept having sex without using a condom when trusting their sexual partner was as high as 1.93 (95%CI 1.44-2.60, p<0.0001, Table 2, column 4, model 4).

Among MSM who are married/live together, trust in the sexual partner was also associated with ICU during last anal intercourse. However, this association was weaker when compared with single/separated/widowed MSM (PRa=1.31, 95%CI 1.11-1.77, p=0.045, Table 2, column 6, model 4).

## DISCUSSION

In this study, we quantitatively test the hypothesis of the association between trust in a sexual partner and ICU among MSM in Brazil. Our results indicate multiple key findings. First, almost half of MSM in our study (n=2,010) had ICU during their last anal intercourse (48.9%, 880). Also, the prevalence of ICU was higher among MSM who said they accept having sex without using a condom when they trust their sexual partner (84.2%). Second, trust in a sexual partner had a strong association with ICU during the last anal intercourse among all MSM in our study. Third, this association remains statistically significant in our stratified analysis among single/separated/widowed MSM and among MSM who are married/live together, indicating that trust can be experienced in different relationship contexts, not only with main or steady partners, such as a husband or boyfriend. Since all these findings were supported by previous literature^13^ ^,^ ^14^ ^,^ ^15^ ^,^ ^17^, they should be better contextualized.

The prevalence of ICU during the last anal intercourse among MSM who accept having sex without using a condom when trusting their sexual partner in our study was higher compared to other Brazilian studies about ICU among MSM with casual partners (50.8%)^18^, commercial partners (26%)^19^, and in a study with 3,449 MSM from 10 Brazilian capitals (36.5%)^6^. It is also higher when compared with other studies with MSM in Chile (43.9% with casual partners)^7^, Sweden (52% with casual partners)^8^, Hong Kong (48.5% with regular partners and 27.3% with non-regular partners)^9^, and Tokyo (37% with regular partners and 18% with casual partners)^20^.

Despite some limitations due to different recruitment approaches and measures of condom use and trust, these comparisons indicate some concerns about ICU among MSM when individuals trust their sexual partners, as lack of condom use remains the main risk factor for HIV infection among MSM worldwide^10^. Also, condoms are a more available prevention strategy against HIV infection, especially in environments marked by significant social inequalities that can limit access to other prevention tools^11^ ^,^ ^12^ ^,^ ^19^. This suggests that MSM in Brazil rarely use even the most accessible prevention strategies. The epidemiological context of HIV infection among MSM in Brazil reflects this scenario since men face an increase in HIV cases, as they represented the majority (65.9%, 450,922 cases) of cases reported between 1991 and September 2025^5^, and evidence from two national studies indicated that HIV prevalence among MSM increased from 10.5% in 2009 to 18.4% in 2016^3^ ^,^ ^4^ ^,^ ^21^.

Echoing the construction of “Safe Sex” in HIV recommendations early in the epidemic, current literature on MSM describes the complex relationship between trust and ICU. Trust was sometimes described as something built over time in a relationship. However, in other studies, it was mentioned as a feeling that was “simply there” and not necessarily related to the duration of the relationship. Trust was also described as the result of explicit or implicit sexual agreements regarding monogamy and, in this setting, was accompanied by feelings of love and intimacy^13^ ^,^ ^15^. In these situations, trust was related to a greater frequency of anal intercourse without a condom, since trustworthy partners are seen as those with lower sexual risk, which allows condoms not to be used^15^.

On the other hand, anal intercourse without a condom was also described as something used to build trust, as MSM stated that not using a condom was proof of their involvement, love, and monogamy with their partner^13^ ^,^ ^15^. In this case, trust was related to difficulties in negotiating prevention behaviors, since talking about condom use with trusted partners, with condomless anal intercourse that had already occurred, could introduce suspicions about betrayal, infidelity, and the partner’s intentions^15^. All these aspects can explain the association we found between trust in the sexual partner and having sex without using a condom and ICU among MSM in our study.

We measured trust by asking the question, “When trusting a sexual partner, do you agree to have sex without using a condom?” Thus, MSM who had a partner, such as a boyfriend or husband, could answer this by considering the partner with whom they already had a relationship. To expand our analysis and evaluate trust in different contexts, we performed a stratified analysis regarding marital status. Even among single/separated/widowed MSM, trust in a sexual partner remains strongly associated with ICU during last anal intercourse. This indicates trust can be experienced in different relationships, not only with main or steady partners, such as a husband or boyfriend. Evidence from previous research supports this, as MSM who report a high frequency of past unprotected anal intercourse are more likely to believe that it is not necessary to use condoms with a trusted or steady partner regardless of their current partnership status^14^.

Among MSM who are married/live together, trust was also associated with a higher prevalence of ICU during last anal intercourse. However, in our study, this association was weaker than among single/separated/widowed MSM. This may be explained by the smaller sample size of MSM who are married/live together in our study (n=263), since the direction of association is supported by previous studies that looked at ICU among MSM with steady partners, such as a boyfriend, a husband, and a loved one^14^ ^,^ ^15^ ^,^ ^17^.

This study expands the existing literature on ICU among Brazilian MSM and analyzes the role of trust in this sexual behavior. However, our analysis has limitations, including potential selection bias related to the networks recruited and limitations in result interpretation when using RDS. The main objective of the study was to conduct biological and behavioral surveillance comparable to that conducted by the WHO and other health authorities. The opportunity to explore the role of trust, lust, faithfulness, or monogamy according to a specific partner type (regular, casual, and commercial partners) was also limited by the available sample size, which was insufficient to conduct reliable, stratified analyses of ICU by partner type. Consequently, we could not assess whether the association between trust and ICU differs according to the type of sexual partner. As discussed in the literature, these aspects may also influence the relationship between trust and ICU^22^.

We measured ICU during the last anal intercourse. This may not be representative of the sexual behavior of MSM but is less subject to recall bias, so comparisons with other studies on condom use among MSM require caution.

Trust plays an important role in sexual behavior among MSM, as it is associated with a higher prevalence of ICU. Given the growing HIV epidemic among MSM in Brazil, HIV prevention strategies should explicitly address the influence of trust on condom use decisions. During counseling, healthcare professionals should encourage discussions about perceived HIV risk within trusting relationships, partners’ HIV status, regular HIV testing, and the adoption of complementary prevention strategies, such as pre-exposure prophylaxis. This approach may help MSM recognize that emotional trust does not eliminate HIV risk and support more informed sexual health decisions.
